# Expression of histone deacetylases 1, 2 and 3 in urothelial bladder cancer

**DOI:** 10.1186/1472-6890-14-10

**Published:** 2014-03-13

**Authors:** Cédric Poyet, Bastian Jentsch, Thomas Hermanns, Daniel Schweckendiek, Hans-Helge Seifert, Martin Schmidtpeter, Tullio Sulser, Holger Moch, Peter J Wild, Glen Kristiansen

**Affiliations:** 1 Department of Urology, University of Zürich, Zürich, Switzerland; 2 Institute of Pathology, University of Zürich, Zürich, Switzerland; 3 Department of Pathology, University of Bonn, Bonn, Germany; 4 Department of Urology, Hegau-Bodensee Hospital, Singen, Germany; 5 Institute of Pathology, University of Bonn, Sigmund-Freud-Str. 25, Bonn D-53127, Germany

**Keywords:** Class I HDACs, Urothelial cancer, Molecular markers

## Abstract

**Background:**

Histone deacetylases (HDACs) are known to be associated with an overexpression in different types of cancer such as colon and prostate cancer. In this study we aimed to evaluate the protein expression of class I HDACs in urothelial carcinoma of the bladder.

**Methods:**

A tissue microarray containing 348 tissuesamples from 174 patients with a primary urothelial carcinoma of the bladder was immunohistochemically stained for HDAC 1, 2 and 3. Intensity of staining was evaluated and the association with clinico-pathological features and prognosis was assessed.

**Results:**

High HDAC expression levels were found in 40 to 60% of all investigated urothelial carcinomas (HDAC-1: 40%, HDAC-2: 42%, HDAC-3: 59%).

HDAC-1 and HDAC-2 were significantly associated with higher tumour grades.

Although all three markers could not predict progression in univariate analyses, high HDAC-1 expression was associated with a trend toward poorer prognosis. Patients with high-grade tumours and high expression levels of HDAC-1 were more likely to progress compared to all other patients (p < 0.05).

**Conclusions:**

High-grade noninvasive papillary bladder tumours are associated with high expression levels of HDAC-1 and HDAC-2. High grade tumours in combination with high expression of HDAC-1 showed a worse prognosis than the other tumours. The high expression levels of HDACs observed particularly in high grade urothelial bladder cancer clearly warrant subsequent studies on the potential use of HDAC inhibitors as a novel therapeutic approach.

## Background

The majority of bladder cancer patients (75-80%) initially present with papillary noninvasive (pTa) or superficially invasive (pT1) urothelial carcinoma, whereas the remaining 20-25% of primary tumours are already muscle invasive (≥ pT2) at first diagnosis [1,2].

Among superficial tumours, almost 70% recur after transurethral resection and up to 25% of them show progression into a muscle invasive disease [3]. Bladder cancer patients have to be monitored closely for disease recurrence and progression, which contributes to the high costs of this disease. Therefore there is a great interest in identifying markers that can diagnose superficial cancer with a high risk of progression and allow for more specific surveillance strategies [4]. So far no established marker allows prediction of tumour progression.

Histone deacetylases (HDACs) constitute a family of enzymes that deacetylate histones and other cellular proteins. They are major regulators of transcription and are also important in other cellular processes [5]. HDACs are classified into four different classes based on the phylogenetic analysis of their structure and homology to yeast enzymes [6]. Class I HDACs are divided into four isoforms (HDAC-1, -2, -3 and -8) and are known to be associated with an overexpression in different types of cancer such as colon and prostate cancer [7,8]. Published expression array data for urothelial cancer could demonstrate an overexpression of different class I HDACs compared to normal urothelium. Especially, the first three isoforms HDAC-1, -2 and -3 were found to be overexpressed. Contrary to HDAC-8, for which no overexpression was found [2,9-12]. In contrast to these findings, a more recent study of Xu and colleagues reported no difference of expression in the expression levels of HDAC-2 between normal urothelial and bladder cancer tissue as assessed by immunohistochemistry [13]. Few studies have found an effect for HDAC-inhibitors (HDAC-i) in urothelial cancer cell lines [12,14-17], however, a broad expression analysis of HDACs in urothelial carcinomas has not been conducted so far. In addition, there is no study available on the prognostic relevance of class I HDACs in bladder cancer. We aimed to analyse the expression patterns of the most promising class I HDACs (HDAC-1, -2 and -3) in a representative cohort of primary bladder cancers and correlated these to clinico-pathological parameters including tumour stage, grade, multifocality, adjacent carcinoma in situ, growth pattern and finally clinical follow-up data.

## Methods

### Bladder cancer tissue microarray (TMA)

Tissue microarrays (TMA) contained 348 formalin-fixed, paraffin-embedded urothelial bladder cancer tissues from 174 patients and were constructed as previously described [18]. All tumour samples were represented in duplicate tissue cores (1 mm). The TMA consisted of tumour tissues only, normal urothelial samples were not available. Specimens were collected between 1990 and 2006 by the Institute of Surgical Pathology, University of Zurich, Switzerland. The TMA includes a series of 174 consecutive (non-selected) primary urothelial bladder tumours. Finally, the TMA contained 90 pTa, 68 pT1 and 16 ≥ pT2 tumours. Hematoxylin and eosin–stained slides of all specimens were reevaluated by two experienced pathologists (BJ, GK).

Tumour stage and grade were assigned according to UICC and WHO criteria.

Retrospective clinical follow-up data were available for all of 174 patients (100%). The median follow-up period for the entire cohort was 110.6 months (range 32.4 to 266.8 months). Clinico-pathologic data are summarized in Table 1. The study was approved by the local scientific ethics committee Kantonale Ethikkommission Zürich (http://www.kek.zh.ch/, approval no.: StV-Nr. 25/2007).

**Table 1:** Patient and tumor characteristics and results of molecular and immunohistochemical analyses

**Variable**	**Categorization**	**n analyzable**	**%**
Total n = 174			
***Clinico-pathologic data:***			
Age at diagnosis (median, range)		69,5 years (32–92)	
	<70 years	87	50.0
	≥70 years	87	50.0
Sex			
	Female	43	24.7
	Male	131	75.3
Tumor stage (WHO 1973^a^)			
	pTa	90	51.7
	pT1	68	39.1
	pT2	13	7.5
	pT3	2	1.1
	pT4	1	0.6
Histologic grade (WHO 1973^a^)			
	G1	44	25.3
	G2	87	50.0
	G3	43	24.7
Histologic grade (WHO 2004^b^)			
	Low grade	101	58.0
	High grade	73	42.0
Adjacent carcinoma in situ			
	No	158	90.8
	Yes	16	9.2
Multiplicity			
	Solitary	124	71.3
	Multifocal	50	28.7
Growth pattern			
	Papillary	159	91.4
	Solid	15	8.6
***Immunohistochemistry (IHC):***			
HDAC-1			
	Low expression	104	59.8
	High expression	70	40.2
HDAC-2			
	Low expression	101	58.0
	High expression	73	42.0
HDAC-3			
	Low expression	71	40.8
	High expression	103	59.2

^a^Staging and grading according to the 1973 WHO classification system.

^b^Staging and grading according to the 2004 WHO classification system.

### Immunohistochemistry

The TMA was freshly cut (3 μm). For immunohistochemical detection of HDAC-1, -2 and -3 isoforms on tissue samples, prediluted polyclonal rabbit IgG antibody directed against HDAC-1 (1:11, Abcam, Cambridge, UK), monoclonal mouse IgG antibody directed against HDAC-2 (1:5000, Abcam) and monoclonal mouse IgG antibody directed against HDAC-3 (1:500, Becton Dickinson, Franklin Lakes, NJ, USA) was used on 3 μm paraffin sections, as described [19]. Ki-67 was detected with clone MIB-1 (Dako, Glostrup, Denmark; dilution 1:50).

Immunohistochemical studies utilised an avidin-biotin peroxidase method with a diaminobenzidine (DAB) chromatogen. After antigen retrieval (microwave oven for 30 min at 250 W) immunohistochemistry was carried out in a NEXES immunostainer (Ventana, Tucson, AZ) following manufacturer’s instructions.

### Evaluation of Immunohistochemistry

One surgical pathologist (BJ) evaluated the slides under the supervision of the senior author. Nuclear staining of HDAC isoforms was scored applying a semiquantitative immunoreactivity scoring (IRS) system that incorporates the percentual area and the intensity of immunoreactivity resulting in a score ranging from 0 to 12, as described previously [19]. For statistical analysis, the intensity of HDAC expression was grouped into low vs. high rates of expression. Cases exhibiting an IRS from 0–8 were pooled in a HDAC low expression group whereas cases with a higher IRS (9–12) were designated HDAC high expression group. The percentage of Ki-67 positive cells of each specimen was determined as described previously [20]. High Ki-67 labelling index was defined as more than 10% of positive tumour cells [21].

### Statistical analysis

Statistical analyses were performed with SPSS version 20.0 (SPSS Inc, Chicago, IL). Differences were considered significant if *p* < 0.05. To study statistical associations between clinicopathologic and immunohistochemical data, contingency table analysis and 2-sided Fisher’s exact tests were used. Univariate Cox regression analysis was used to evaluate statistical association between clinicopathologic/immunohistochemical data and progression free survival (PFS). PFS curves were calculated using the Kaplan–Meier method with significance evaluated by 2-sided log-rank statistics. For the analysis of PFS, patients were censored at the date when there was a stage-shift (from Ta to T1-4, respectively from T1 to T2-T4), or if there was distant metastatic disease.

## Results

### Staining patterns of HDAC1-3

**Figure 1:**
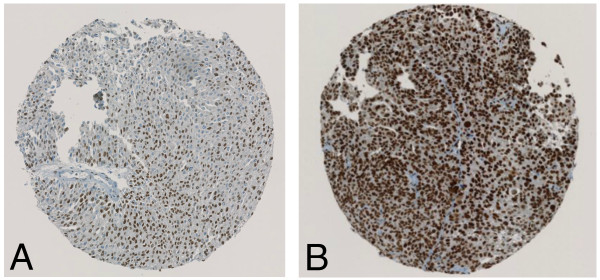
**Immunohistochemical staining with HDAC-1. (A)** low expression HDAC-1 staining pattern in a low-grade urothelial tumour; **(B)** high expression HDAC-1 staining pattern in a typically high-grade urothelial tumour.

**Figure 2:**
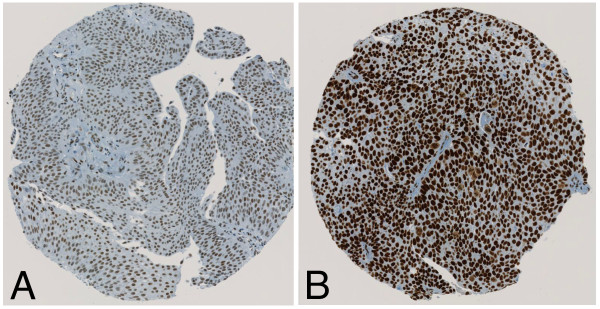
**Immunohistochemical staining with HDAC-2. (A)** low expression HDAC-2 staining pattern in a low-grade urothelial tumour; **(B)** high expression HDAC-2 staining pattern in a typically high-grade urothelial tumour.

**Figure 3:**
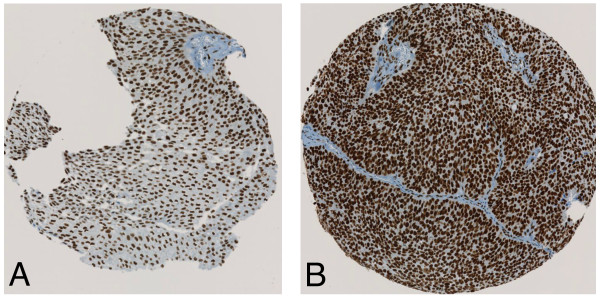
**Immunohistochemical staining with HDAC-3. (A)** low expression HDAC-3 staining pattern in a low-grade urothelial tumour; **(B)** high expression HDAC-3 staining pattern in a typically high-grade urothelial tumour.

HDAC 1–3 protein expression in bladder cancer tissue samples was investigated by immunohistochemical analysis of the TMA containing 174 specimens from patients with a primary urothelial carcinoma of the bladder. All 174 (100%) patients could be evaluated for HDAC immunostaining. All three investigated HDACs showed high expression levels in 40 to 60% of all tumours. Figures 1, 2 and 3 represent examples of typical exclusively nuclear staining patterns of HDAC-1, -2 and -3 (low - and high expression). For HDAC-1 40% of the tumours showed high expression levels, for HDAC-2 42% and for HDAC-3 even 59% (Table 1).

### Correlations to clinico-pathological parameters

HDAC-1 to 3 and Ki-67 were correlated with clinico-pathologic characteristics (stage, grading, adjacent carcinoma in situ, multiplicity and growth pattern) of the tumours (Table 2). Strong staining of HDAC-1 and HDAC-2 was associated with higher grading (both WHO 1973 and 2004), additionally tumours with high expression levels of HDAC-2 presented more often with adjacent carcinoma in situ compared to tumours with weak HDAC-2 staining. High expression levels of HDAC-3 were only associated with higher tumour grade according the new WHO 2004 grading system. Ki-67 showed a significant correlation with all clinico-pathologic characteristics (p < 0.05), except for tumour multiplicity. The expression levels of all three tested HDAC proteins were significantly associated with each other (data not shown). Furthermore, strong staining of all three HDACs correlated with high Ki-67 labelling index (for HDAC-1: Spearmans Rho *r*_*s*_ = 0.325, p < 0.001, for HDAC-2: *r*_*s*_ = 0.271, p < 0.001; for HDAC-3: *r*_*s*_ = 0.191, p < 0.05).

**Table 2:** Associations of the HDAC-1, -2 and 3 and Ki-67 with pathologic characteristics (n = 174)

**Variable**	**Categorization**	**HDAC-1 expression**			**HDAC-2 expression**			**HDAC-3 expression**			**Ki-67 expression**		
		**Low**	**High**	**p**	**Low**	**High**	**p**	**Low**	**High**	**p**	**Low**	**High**	**p**
Tumor stage (WHO 1973)^a^													
	pTa	59	31	0.162	55	35	0.811	37	53	0.257	71	19	**<0.001**
	pT1	37	31		37	31		30	38		35	33	
	pT2	5	8		7	6		2	11		2	11	
	pT3	2	0		1	1		1	1		0	2	
	pT4	1	0		1	0		1	0		0	1	
Histologic grade (WHO 1973)^a^													
	G1	29	15	**0.022**	32	12	**0.026**	20	24	0.425	39	5	**<0.001**
	G2	57	30		50	37		37	50		61	26	
	G3	18	25		19	24		14	29		8	35	
Histologic grade (WHO 2004)^b^													
	Low grade	71	30	**0.001**	68	33	**0.005**	49	52	**0.019**	87	14	**<0.001**
	High grade	33	40		33	40		22	51		21	52	
Adjacent carcinoma in situ^b^													
	No	96	62	0.432	96	62	**0.032**	65	93	1.00	105	53	**<0.001**
	Yes	8	8		5	11		6	10		3	13	
Multiplicity^b^													
	Solitary	79	45	0.124	77	47	0.093	49	75	0.587	86	38	**0.003**
	Multifocal	25	25		24	26		22	28		22	28	
Growth pattern^b^													
	Papillary	97	62	0.278	94	65	0.416	65	94	1.00	107	52	**<0.001**
	Solid	7	8		7	8		6	9		1	14	

^a^Chi-Square Pearson (2-sided); bold face representing p-values < 0.05.

^b^Fisher’s exact test (2-sided); bold face representing p-values < 0.05.

### Univariate progression analyses

A total of 158 patients underwent TUR for a primary Ta or T1 urothelial carcinoma of the bladder and were followed for a median of 110.7 month (range: 32.4 - 245.9 month). In this group, only high expression levels of Ki-67 were significantly associated with increased risk of progression (p < 0.01). Increased expression of HDAC-1 showed a tendency for higher progression rates, however this was not statistically significant (p = 0.085). Beside growth pattern none of the clinicopathological parameters were associated with PFS. Table 3 shows *p*-values for the pathological data and the molecular markers.

**Table 3:** Univariate analyses of disease progresssion (n =158)

**Variable**	**Categorization**	**Tumor progression**		
		**n**^**a**^	**events**	**p**^**b**^
***Pathologic data:***				
Tumor stage (WHO 1973^c^)				
	pTa	85	10	0.412
	pT1	68	12	
Histologic grade (WHO 1973^c^)				
	G1	43	3	0.093
	G2	82	12	
	G3	28	7	
Histologic grade (WHO 2004^d^)				
	Low grade	95	10	0.092
	High grade	58	12	
Adjacent carcinoma in situ				
	No	141	20	0.570
	Yes	12	2	
Multifocality				
	Unifocal tumor	111	15	0.486
	Multifocal tumor	42	7	
Growth pattern				
	Papillary	146	17	**<0.0001**
	Solid	7	5	
***Immunohistochemistry:***				
HDAC-1				
	Low expression	92	9	0.085
	High expression	61	13	
HDAC-2				
	Low expression	88	12	0.628
	High expression	65	10	
HDAC-3				
	Low expression	65	8	0.754
	High expression	88	14	
Ki-67 IHC				
	Low expression	101	9	**0.004**
	High expression	52	13	

^a^Only primary pTa and pT1 tumours are included.

^b^Log Rank test (2-sided); bold face representing p-values <0.05.

^c^Staging and grading according to the 1973 WHO classification system.

^d^Staging and grading according to the 2004 WHO classification system.

To test whether the combination of high expression levels of HDAC-1 & HDAC-2 with different known clinico-pathological parameters can predict prognosis we performed an univariate cox-regression analysis. The combination of high-grade tumours and high expression levels of HDAC-1 was a predictor of PFS (hazard ratio [HR], 1.640; 95% confidence interval [95% CI], 1.021-2.636; p = 0.044). However, this combination did not outperform tumor growth pattern or Ki-67 as a predictor of outcome. Both parameters were able to predict prognosis in univariate analysis (Table 3). The combination of HDAC-1 and growth pattern or HDAC-1 and Ki-67 were of no additional value to predict prognosis. In both cases the p-values were higher than for growth pattern or Ki-67 alone (data not shown).

**Figure 4:**
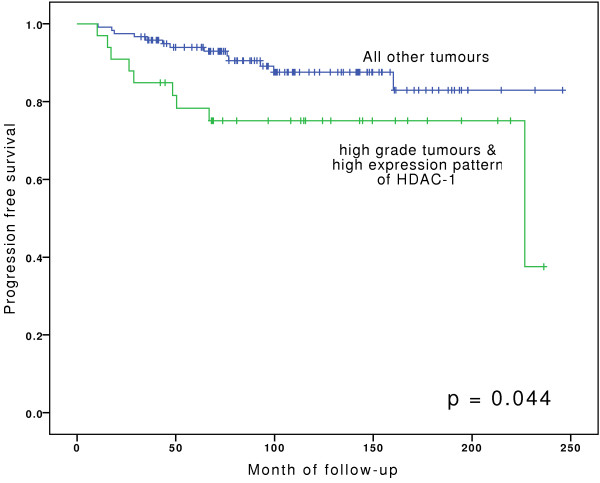
**Kaplan-Meier analyses for progression-free survival for group of high-grade tumours in combination with high expression levels of HDAC-1 vs. high- and low-grade tumours with low expression levels of HDAC-1.** For statistical analysis for survival curves Log-Rank test was used.

**Figure 5:**
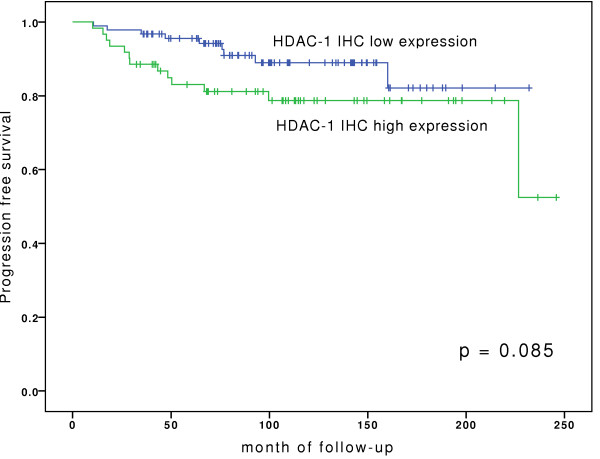
**Kaplan-Meier analyses for progression-free survival for HDAC-1 staining.** For statistical analysis for survival curves Log-Rank test was used.

**Figure 6:**
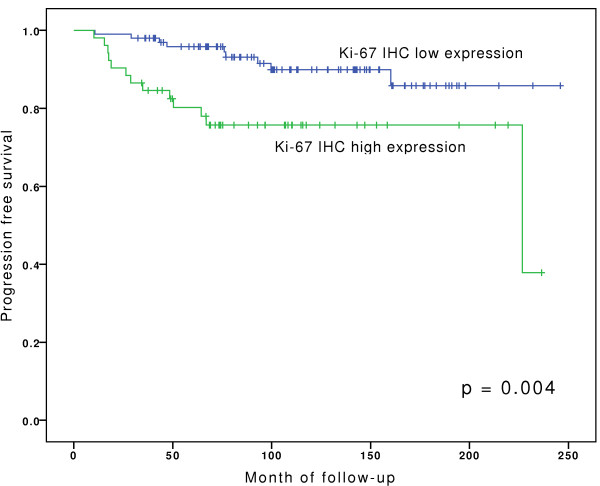
**Kaplan-Meier analyses for progression-free survival for ki-67 staining.** For statistical analysis for survival curves Log-Rank test was used.

Kaplan-Meier analyses for PFS are depicted in Figures 4, 5, 6, and show that bladder cancer patients with the combined feature of high grade tumours and high expression pattern of HDAC-1 have a significantly shorter progression free survival than all other patients (Figure 4). High HDAC-1 expression alone showed a tendency for shorter PFS, although not statistically significant (Figure 5). In addition, patients with high expression levels of Ki-67 have a significantly shorter PFS (Figure 6).

## Discussion

This is the first comprehensive immunohistochemical analysis of the expression of several class I HDAC proteins (1, 2 and 3) in urothelial carcinoma. In our study, we found all three isoforms in a relevant amount of all investigated urothelial tumours. HDAC-1 and HDAC-2 were highly associated with high-grade superficial papillary bladder tumours. Additionally, high expression levels of HDAC-1 showed a tendency towards a shorter PFS.

So far, little was known about class I HDAC expression pattern in urothelial cancer [5]. According to the Proteinatlas (http://www.proteinatlas.org), HDAC-1 to -3 expression levels are moderate at most in urothelial cancer [22]. In previous expression arrays HDAC-2 and -3 showed higher expression levels in urothelial cancer than in normal urothelial tissue [2,9,11]. Expression array data from another study by Wild et al. demonstrated an upregulation of HDAC-1 in bladder cancer compared to normal urothelial tissue [23]. On the contrary, published data from other groups did not reveal any difference of class I HDAC expression between urothelial cancer and normal urothelium in microarray data [24,25]. In accordance with these findings a study from Xu reported no difference in immunohistochemical expression of HDAC-2 in human bladder cancer tissue (142 cases) compared to normal urothelial tissue (23 cases) [13].

In a recent study, Niegisch and colleagues were able to show upregulation of HDAC-2 mRNAs in a subset of tested tumours compared to normal urothelium [9,11,17]. However, only 24 tumour tissues and 12 normal samples were tested.

Our study is the first attempt to test the immunohistochemical expression of class I HDACs in a large cohort of patients with bladder cancer. As class I HDACs can be detected in a relevant group of urothelial cancer, they may therefore be relevant in pathophysiology and as target proteins for treatment.

Besides the distinct presence of class I HDACs in urothelial cancer, high expression levels of HDAC-1 and -2 were associated with stage and grade of this tumours. Overexpression of HDAC*s* has been found in several other solid tumours such as prostate and colon cancer [7,26,27]. High expression levels of class I HDACs correlated with tumour dedifferentiation and higher proliferative fractions (measured by Ki-67) in urothelial carcinoma, which is in line with *in vitro* studies showing that high HDAC activity leads to tumour dedifferentiation and enhanced tumour cell proliferation [28-30]. Despite the growth inhibitory effects of HDAC-i demonstrated in various cell lines including bladder cancer cells, a broad expression analysis of this attractive target has not been conducted yet [12-16,31,32].

To the best of our knowledge, this is the first study analysing HDAC-1, -2 and -3 expression in bladder cancer and its association to prognosis. In our study HDAC-1 was found to be of rough prognostic relevance in pTa and pT1 tumours. High expression levels of class I HDACs have been found to be of prognostic relevance in other tumour entities before. Other study groups previously reported the association of class I HDACs with more aggressive tumours and even shortened patient survival in prostate and gastric cancer [7,33]. Our findings suggest that HDAC-1 may have a role in prognosis of superficial urothelial tumours.

In our work the rate of Ki-67 positive tumour cells was highly associated with tumour grade, stage, and a shorter PFS. A substantial amount of research has demonstrated the prognostic role of Ki-67 in urothelial cancer; its prognostic value and its association with pathological parameters and prognosis could be shown in several studies [34-36]. These findings are in line with our work and confirm the representativeness and validity of this TMA-construct. Furthermore, we observed a strong correlation between the proliferation index (Ki-67) and all three investigated HDACs. The connection between HDAC expression and Ki-67 observed in urothelial carcinoma has already been demonstrated for prostate, renal and colorectal cancer in previous studies [7,19,37].

Additionally, intravesical instillation of HDAC-i may have a potential as chemopreventive agent to treat superficial bladder cancer, as up to 50% of superficial tumours showed high expression levels of HDACs. However, it is not clear whether HDAC protein expression as assessed by immunohistochemistry is a predictor for treatment response to HDAC-i. Thus, additional studies are needed to clarify the role HDAC-i in non-invasive urothelial cancer.

Our study has several limitations, including its retrospective design and the use of immunohistochemical methodology, which has inherent limitations, including scoring of staining. We used a standardized and well-established semiquantitative scoring method in accordance with previous publications to reduce variability. In addition, the proportion of muscle-invasive bladder cancer was limited and as a consequence we cannot draw any conclusion for this subgroup of tumours. Therefore future research should also try to assess whether class I HDACs have a prognostic value in locally advanced invasive or metastatic urothelial cancer.

## Conclusion

High levels of class I HDACs showed a significant correlation with cellular proliferation and tumor grade. Non-invasive (pTa) and pT1 bladder tumours with high expression levels of HDAC-1 showed a tendency towards shorter PFS in our cohort. However, further prospective studies and bigger cohorts including muscle-invasive bladder cancer patients are needed to evaluate the prognostic value of HDACs. Moreover the high expression levels of HDACs in urothelial bladder cancer might be indicative for a treatment response to HDAC-i which ought to be evaluated in further studies.

## Abbreviations

HDACs: Histone deacetylases; HDAC-i: HDAC-inhibitors; TMA: Tissue microarray; IRS: Immunoreactivity scoring; PFS: Progression free survival.

## Competing interests

The authors declare that they have no competing interests.

## Authors’ contributions

CP coordinated the study, performed statistical analyses and wrote the paper. BJ performed immuno-histological analyses and wrote the paper. TH, HHS, TS coordinated the study and revised essential parts of the paper. CP, DS and MS provided clinicopatholocial data for the study. PJW and HM supported the study coordination, assisted in technical questions and statistical support. GK conceived and coordinated the study, performed statistical analyses, wrote and revised the paper. All authors read and approved the final manuscript.

## Pre-publication history

The pre-publication history for this paper can be accessed here:

http://www.biomedcentral.com/1472-6890/14/10/prepub
